# Retraction: Commentary: Schema therapy for Dissociative Identity Disorder: a case report

**DOI:** 10.3389/fpsyt.2024.1473694

**Published:** 2024-08-02

**Authors:** Frontiers Editorial Office

*The journal retracts the 28 February 2024 article cited above.*

Following publication, the Editorial Office was made aware that an undeclared conflict of interest existed for this manuscript. This lack of declaration was an oversight by the authors but impacted the editors’ fair assessment of the manuscript as well as the peer review process.

An investigation was conducted in accordance with Frontiers’ policies that confirmed this; therefore, the article has been retracted. The authors agree to this retraction.

